# Disordered eating behaviours and their associated factors in adults with type 2 diabetes: A multicentre cross-sectional study

**DOI:** 10.51866/oa.1158

**Published:** 2026-07-30

**Authors:** Yong Xian Goo, Cheau Foong Fam, Vithiyasri Nithiyanandan, Siti Noorul Ain Abdul Jalil, Norshafika Norlaili, Khausar Hazman, Yen Sue Quay, Subashini Ambigapathy, Siew Mooi Ching, Hooi Min Lim

**Affiliations:** 1 Department of Primary Care Medicine, Faculty of Medicine, Universiti Malaya, Kuala Lumpur, Malaysia.; 2 Bandar Botanic Health Clinic, Ministry of Health Malaysia, Klang, Selangor, Malaysia.; 3 Seksyen 19 Health Clinic, Ministry of Health Malaysia, Shah Alam, Selangor, Malaysia.; 4 Jenjarom Health Clinic, Ministry of Health Malaysia, Jenjarom, Selangor, Malaysia.; 5 Sungai Bertek Health Clinic, Ministry of Health Malaysia, Klang, Selangor, Malaysia.; 6 Buntong Health Clinic, Ministry of Health Malaysia, Ipoh, Perak, Malaysia.; 7 Department of Family Medicine, Faculty of Medicine and Health Sciences, Universiti Putra Malaysia, Serdang, Selangor, Malaysia.

**Keywords:** Feeding and Eating Disorders, Eating Behavior, Diabetes Mellitus, Type 2, Anxiety

## Abstract

**Introduction:**

Dietary and weight control in type 2 diabetes (T2D) can place psychological strain on patients, increasing the risk of disordered eating behaviours (DEBs). This study aimed to determine the level of DEBs using the validated Eating Disorder Examination Questionnaire (EDE-Q) and its associated sociodemographic, clinical and mental health factors in adults with type 2 diabetes in Malaysia.

**Methods:**

We conducted a multicentre cross-sectional study in four government primary care clinics among 412 patients with T2D. DEBs were assessed using the Malay EDE-Q 6.0, while depression and anxiety were evaluated using the Patient Health Questionnaire-9 and Generalised Anxiety Disorder-7 (GAD-7), respectively. Multivariable linear regression was performed on the square-root transformed EDE-Q global score to identify independent associated factors.

**Results:**

The mean EDE-Q global score was 1.01±1.09, with shape concern being the highest- scoring subscale. Higher DEB levels were associated with younger age (B=–0.01, 95% confidence interval [CI]=–0.02 to –0.01, P<0.001), higher body mass index (BMI) (B=0.02, 95% CI=0.02 to 0.03, P<0.001), higher GAD-7 score (B=0.05, 95% CI=0.03 to 0.08, P<0.001) and higher educational level (secondary [B=0.21, 95% CI=0.07 to 0.35, P=0.003] and tertiary education [B=0.31, 95% CI=0.13 to 0.48, P<0.001]). The Chinese participants had lower EDE-Q scores than the Malay participants (B=–0.18, 95% CI=–0.31 to –0.04, P=0.012).

**Conclusion:**

Overall DEB level is low, however, risks are higher among patients who are younger, are more educated, have a higher BMI and have greater anxiety. Targeted identification of these high-risk groups facilitates early interventions to prevent adverse complications.

## Introduction

Effective glycaemic control of type 2 diabetes relies on sustained self-care behaviours such as selfregulation of eating, adherence to dietary modification and weight control.^1^ The emphasis on strict eating patterns and body weight regulation may place psychological strain on some people, unintentionally increasing vulnerability to maladaptive eating-related behaviours.^2^

Eating disorders (EDs) are psychiatric conditions characterised by a persistent disturbance of eating or eating-related behaviours that result in the altered consumption or absorption of food and significantly impair physical health or psychosocial functioning.^3^ While the clinical diagnosis of EDs such as anorexia nervosa or binge eating is well delineated, it is also important to recognise disordered eating behaviours (DEBs). DEBs are a broader descriptive term for maladaptive eating patterns that do not necessarily meet the full frequency or severity thresholds for a formal ED diagnosis.^4^

In type 2 diabetes, DEBs can severely compromise glycaemic control and long-term health outcomes through mechanisms such as compensatory eating to avoid hypoglycaemia, binge eating and fluctuations driven by stress–eating cycles.^5^ Weight stigma in diabetes care and the psychological burden of chronic disease management can also exacerbate these pathologies.^6^ Integrating clinical awareness of DEBs into routine care helps identify hidden psychological barriers that can lead to poor treatment adherence. Early detection of these patterns is a proactive strategy to prevent longterm diabetes complications and maintain stable metabolic health.

While the association between type 1 diabetes and EDs is well established,^7^ research examining the relationship between type 2 diabetes and DEBs is limited.^5^ Studies investigating EDs have predominantly focused on populations such as adolescents, university students and the general population,^8^ leaving a significant data gap regarding the population with type 2 diabetes. There is limited examination of psychological depression and anxiety and their clinical correlation with DEBs. Data regarding DEBs in patients with type 2 diabetes are predominantly from Western populations,^4,9^ with underrepresentation of Asian and low-to-middle-income countries. Limited evidence accurately reflects the sociocultural influences, dietary patterns and Asian-specific body mass index (BMI) profiles in low-to-middle-income Asian countries. This underrepresentation leaves a significant data gap in understanding how DEBs manifest in a multi-ethnic Asian population. Identifying the level of DEBs and its associated factors among people with type 2 diabetes is essential to improve clinical screening and optimise diabetes management protocols. The aim of this study was to determine the level of DEBs using the Eating Disorder Examination Questionnaire (EDE-Q) and its association with relevant sociodemographic, clinical and mental health factors in adults with type 2 diabetes in Malaysia.

## Methods

### Study design and setting

We conducted a multicentre cross-sectional study in four government primary care clinics in Selangor, Malaysia, covering urban (Seksyen 19 Shah Alam, Bandar Botanic and Sungai Bertek) and rural areas (Jenjarom), from February to September 2023.

### Study participants and sampling

We recruited patients who were aged ≥18 years, had a diagnosis of type 2 diabetes and attended the selected clinics during the study period. We excluded patients who had type 1 diabetes, were pregnant, had cognitive impairments, were unable to read Malay or English or were too unwell to participate. There was no minimum duration of diabetes required for eligibility, and being registered in the National Diabetes Registry was not an inclusion criterion, although the majority of participants were enrolled therein.

Participants were recruited using a convenience sampling method, wherein they were approached at the clinic triage counter at their convenience. We calculated the sample size based on twotailed α coefficient of 0.05, power of 80% and estimated prevalence of EDs of 40% in people with type 2 diabetes from a similar study.^10^ The sample size calculated was 369 participants. We inflated the number to an additional 10% considering the possibility of non-respondents. The final sample size was 406 participants.

### Study instruments

We used a paper-based, self-administered questionnaire consisting of five sections. Section A captured the following sociodemographic characteristics: age, sex, marital status, ethnicity, educational level, occupation, locality and monthly household income. The official threshold for the bottom 40% (B40) income group varies across different states in Malaysia; this study adopted a simplified, approximate cutoff of RM 4850 (equivalent to approximately 1000 USD) to standardise household income categorisation for analysis. Section B captured clinical characteristics and comorbidities including duration of diabetes, current treatment regimen (i.e. oral hypoglycaemic agents and/or insulin) and concurrent medical conditions. These data were self-reported and verified against participants’ medical records.

Section C assessed mental well-being using the Patient Health Questionnaire-9 (PHQ-9) and Generalised Anxiety Disorder-7 (GAD-7). The PHQ-9 consists of nine items assessing depressive symptoms over the past 2 weeks, with scores ranging from 0 to 27. PHQ-9 scores of 5, 10, 15 and 20 represent cutoffs for mild, moderate, moderately severe and severe depression, respectively. For bivariate analysis, participants were categorised as having depressive symptoms when they scored ≥5. The Malay version of the PHQ-9 was found to have good internal reliability (Cronbach’s a=0.700).^11^ Conversely, the GAD-7 consists of seven items measuring anxiety symptoms, with scores ranging from 0 to 21. For bivariate analysis, participants were categorised as having anxiety symptoms when they scored ≥5. The Malay version of the GAD-7 was also found to have good internal reliability (Cronbach’s α=0.74).^12^

Section D included the EDE-Q 6.0, a 28-item self-report tool measuring DEBs over the preceding 28 days.^13^ It comprises four subscales: restraint, eating concern, weight concern and shape concern. Items are rated on a 7-point scale (0–6), and a global score is calculated by averaging the four mean subscale scores. The Malay version of the EDE-Q 6.0 registered excellent internal reliability (α=0.93), and the test–retest reliability indicated a strong correlation (r=0.83, P<0.01).^14^ Section E comprised a data collection pro forma, which we completed. Weight and height were measured using a validated digital scale to calculate BMI. Clinical data including diabetes medications and the latest glycated haemoglobin (HbA1c) level were captured from medical records.

A pilot study was conducted among 36 patients with type 2 diabetes to face-validate the questionnaire and evaluate the data collection flow. Data from the pilot study were excluded from the final analysis.

### Data collection

Participants were recruited at the triage counters of the selected clinics. Data collection was conducted concurrently across all four primary care centres using a standardised protocol. We screened the personal medical records of attending patients at the triage counters to identify eligible individuals. Eligible participants were approached, provided with an information sheet and given an opportunity to ask questions. Written informed consent was obtained prior to enrolment. Participants completed the self-administered questionnaire while waiting for their consultation. Clinical data, including HbA1c levels, were retrieved from participants’ medical records; therefore, the timing of these measurements was not standardised. This project was approved by the National Medical Research and Ethics Committee (NMRR ID-22-02627-LBB).

### Data analysis

All statistical analyses were conducted using the IBM SPSS Statistics for Windows, version 29.0 (IBM Corp., Armonk, NY, USA). Descriptive data were used to describe participants’ demographic and clinical data, including their PHQ-9, GAD-7 and EDE-Q 6.0 scores. Continuous data were summarised as means and standard deviations (SDs) for normally distributed data or medians and interquartile ranges for skewed data. Categorical data were presented as frequencies and percentages.

For statistical analysis, BMI categories were regrouped into four tiers to ensure sufficient group sample sizes and valid statistical comparisons. These analytical categories used cutoffs as follows: underweight and normal (≤22.9 kg/m^2^), overweight (23-24.9 kg/m^2^), obese I (25-29.9 kg/m^2^) and obese II (≥30 kg/m2). Bivariate analyses were performed to explore associations with the EDE-Q global score using independent samples t-tests (for two-group variables) and one-way analysis of variance (ANOVA) (for variables with three or more groups). The assumption of homogeneity of variances was assessed using Levene’s test. Due to the violation of this assumption (as indicated by Levene’s test P<0.05 for all tested variables), the Games-Howell post hoc test was employed for all significant one-way ANOVA results.

We used multiple linear regression to adjust for confounding variables, including age, BMI, PHQ-9 score, GAD-7 score, household income, sex, educational level, locality and ethnicity. The dependent variable (EDE-Q global score) violated the assumption of homoscedasticity, meaning the spread or variance of the data points was uneven across the model. To correct this inconsistent data spread, we applied a square-root transformation to the EDE-Q global score to meet the assumption of homoscedasticity. This transformed variable was used as the dependent variable in the regression model. Independent variables with a P-value of <0.05 in the bivariate analyses were included in the multivariable linear regression model using the ‘Enter’ method. Results were reported as unstandardised coefficients (B) with 95% confidence intervals (CIs).

## Results

A total of 412 patients were recruited in the study. The mean age of the participants was 56.27±10.66 years (Table 1). The majority were women (61.9%). The participants were predominantly Malay (40.3%) and Indian (40%), followed by Chinese (17%). Most participants (57%) had attained secondary education, and 83% belonged to the B40 monthly household income category. The mean duration of diabetes was 7.79±6.01 years, with a mean HbAlc level of 8.21±1.96%. The mean BMI was 29.10±5.59 kg/m2, with 89.8% of the participants classified as overweight or obese. The majority of the participants reported normal PHQ-9 (87.9%) and GAD-7 (92.7%) scores.

**Table 1:** Sociodemographic and clinical characteristics of the participants (N=412).

Variable	n (%)	Mean ± SD
Age (year)		56.27±10.66
Sex		
Men	157 (38.1)	
Women	255 (61.9)	
Marital status		
Single	22 (5.3)	
Married	327 (79.4)	
Divorced	11 (2.7)	
Widowed	52 (12.6)	
Ethnicity		
Malay	166 (40.3)	
Chinese	70 (17.0)	
Indian	165 (40.0)	
Others	11 (2.7)	
Educational level		
No formal education	13 (3.2)	
Primary education	60 (14.6)	
Secondary education	235 (57.0)	
Tertiary education	104 (25.2)	
Location		
Urban	301 (73.1)	
Rural	111 (26.9)	
Monthly household income		
<RM 4850 (USD 1000)	342 (83.0)	
>RM 4850 (USD 1000)	70 (17.0)	
**Clinical characteristic**		
Duration of diabetes (year)		7.79±6.01
HbA1c level (%)		8.21±1.96
Diabetes medications		
Diet control only	2 (0.5)	
OHA	257 (62.4)	
Insulin injection	9 (2.2)	
Combination of OHA and insulin	144 (35.0)	
BMI (kg/m^2^)		29.10+5.59
Underweight and normal (<22.9)	42 (10.2)	
Overweight (23–24.9)	56 (13.6)	
Obese I (25-29.9)	154 (37.4)	
Obese II (≥30)	160 (38.8)	
Presence of other comorbidities		
Hypertension	300 (72.8)	
Hyperlipidaemia	305 (74.0)	
Heart disease	43 (10.4)	
Stroke	11 (2.7)	
**Mental health variable**		
Depression (PHQ-9 score)		
Normal (0-4)	362 (87.9)	
Mild (5-9)	36 (8.7)	
Moderate (10-14)	10 (2.4)	
Moderately severe (15-19)	3 (0.7)	
Severe (20-27)	1 (0.2)	
Anxiety (GAD-7 score)		
Normal (0-4)	382 (92.7)	
Mild (5-9)	21 (5.1)	
Moderate (10-14)	6 (1.5)	
Severe (15-21)	3 (0.7)	

Data are presented as means ± SDs or frequencies (percentages). SD: standard deviation, BMI: body mass index, HbAlc: glycated haemoglobin, OHA: oral hypoglycaemic agent, RM: ringgit Malaysia, USD: United States dollar.

### EDE-Q global score

The mean EDE-Q global score among the sample was 1.01 (SD=1.09). Among the subscales, shape concern showed the highest mean score (M=1.21, SD=1.44), while eating concern had the lowest mean score (M=0.58, SD=0.90) (Table 2).

**Table 2:** Mean EDE-Q global and subscale scores and their SDs (N=412).

EDE-Q	Mean (SD)
Restraint	1.14 (1.3)
Eating concern	0.58 (0.9)
Shape concern	1.21 (1.44)
Weight concern	1.11 (1.33)
Global score	1.01 (1.09)

Values are presented as means (SDs). Higher scores indicate a greater level of disordered eating behaviours. EDE-Q: Eating Disorder Examination Questionnaire, SD: standard deviation.

### Factors associated with the EDE-Q global score

The bivariate analyses (Table 3) indicated that the EDE-Q global scores were significantly higher among the female (mean difference [MD]=0.22, 95% CI=0.01 to 0.43, P=0.040) and younger participants (P<0.001). The Games–Howell post hoc test for age indicated that the participants aged <50 and 50–59 years (assigned to post hoc group a) had significantly higher EDE-Q global scores than those aged 60–69 and ≥70 years (assigned to post hoc group *b*). The letter designations (*a, b* and *c*) represent statistical subgroups; any two clinical characteristics sharing the same letter do not differ significantly from one another, whereas groups that do not share a common letter are statistically significantly different (P<0.001). The EDE-Q global scores also increased with a higher educational level (P<0.001); the Games–Howell post hoc test revealed significant differences between all three educational groups (groups *a*, *b* and *c*). The urban residents had higher EDE-Q global scores than the rural residents (MD=0.44, 95% CI=0.21 to 0.68, P<0.001), as did those in the high-income group than in the low-income group (MD=0.40, 95% CI=0.12 to 0.68, P<0.001). The Malay and Indian (group *a)* participants had higher EDE-Q global scores than the Chinese participants (group b) (P<0.001). A higher BMI was associated with a higher EDE-Q global score (P<0.001), with the post hoc tests showing that the participants in the obese I (group b) and obese II (group c) categories scored higher than those in the underweight/normal and overweight (group a) categories. The presence of depressive (MD=–0.63, 95% CI=–0.99 to –0.26, P<0.001) and anxiety symptoms (MD=1.06, 95% CI=0.54 to 1.58, P<0.001) was associated with a significantly higher EDE-Q global score.

**Table 3:** Bivariate analyses of the factors associated with the EDE-Q global score (N=412).

Variable	EDE-Q global score, mean (SD)	n	Post hoc group	Mean difference (95% CI)	P-value
Sex					
Men	0.87 (1.01)	157		-0.22 (-0.43 to -0.01)	0.040
Women	1.10 (1.13)	255			
Age (year)					<0.001
<50	1.45 (1.13)	100	*a*		
50-59	1.14 (1.08)	147	*a*		
60-69	0.69 (0.97)	126	*b*		
≥70	0.42 (0.84)	39	*b*		
Ethnicity					<0.001
Malay	1.19 (1.13)	166	*a*		
Chinese	0.49 (0.63)	70	*b*		
Indian	1.06 (1.15)	165	*a*		
Others	0.97 (0.91)	11	*a, b*		
Educational level					<0.001
No formal/primary education	0.42 (0.66)	73	*a*		
Secondary education	1.04 (1.16)	235	*b*		
Tertiary education	1.35 (1.00)	104	*c*		
Location					<0.001
Urban	1.13 (1.15)	301		0.44 (0.21 to 0.68)	
Rural	0.69 (0.84)	111			
Monthly household income					<0.001
Low (<USD 1000)	0.94 (1.08)	342		-0.40 (-0.68 to -0.12)	
High (≥USD 1000)	1.34 (1.08)	70			
HbA1c level (%)					0.184
≤6.5	0.88 (1.03)	74			
6.6-7.5	0.89 (1.05)	111			
7.6-9.0	1.16 (1.07)	117			
≥9.1	1.06 (1.17)	110			
Insulin use				0.01 (-2.14 to 0.22)	0.962
No	1.01 (1.09)	259			
Yes	1.01 (1.10)	153			
Body mass index (kg/m^2^)					<0.001
Underweight and normal (≤22.9)	0.41 (0.53)	42	*a*		
Overweight (23-24.9)	0.49 (0.77)	56	*a*		
Obese I (25-29.9)	0.91 (1.00)	154	*b*		
Obese II (≥30)	1.44 (1.21)	160	*c*		
Comorbidities				0.27 (-0.15 to 0.69)	0.200
Absence	1.25 (1.34)	44			
Presence	0.98 (1.05)	368			
PHQ-9 score				-0.63 (-0.99 to -0.26)	<0.001
Normal (<5)	0.93 (1.05)	362			
Depression (≥5)	1.56 (1.22)	50			
GAD-7 score				-1.06 (-1.58 to -0.54)	<0.001
Normal (<5)	0.933 (1.03)	382			
Anxiety (≥5)	1.99 (1.36)	30			

Data are presented as means (SDs) unless otherwise specified. P-values were derived from independent samples t-tests for two-group variables and oneway ANOVA for variables with three or more groups. The post hoc group letters represent the results of the Games–Howell post hoc tests performed after obtaining significant one-way ANOVA results. Groups that share a common letter are not significantly different from each other (P>0.05). Groups that do not share a common letter are significantly different from each other (e.g. for age, group *a* is significantly different from group b but not from other groups labelled *a*). The full numerical results are available in Supplementary Tables S1–S4. ANOVA: analysis of variance, CI: confidence interval, EDE-Q: Eating Disorder Examination Questionnaire, SD: standard deviation.

### Multiple linear regression of the factors associated with DEBs

Table 4 shows the multivariable linear regression examining the factors associated with DEBs. The EDE-Q global score was square-root transformed as the dependent variable to meet the assumption of homoscedasticity. The final model was significant (F [12, 399] = 17.318, P<0.001) and explained 32.3% of the variance in the square root of the EDE-Q global score.

**Table 4:** Multiple linear regression of the factors associated with the EDE-Q global score.

Variable	B	P	95% CI	P-value
(Constant)	0.29		-0.20 to 0.78	0.251
Age	-0.01	-0.18	-0.02 to -0.01	<0.001
BMI	0.02	0.23	0.02 to 0.03	<0.001
PHQ-9 score	0.01	0.03	-0.01 to 0.02	0.599
GAD-7 score	0.05	0.24	0.03 to 0.08	<0.001
Household income (non-B40 vs B40)	0.11	0.08	-0.01 to 0.23	0.083
Sex (women vs men)	0.08	0.07	-0.02 to 0.17	0.101
Educational level (secondary vs primary)	0.21	0.19	0.07 to 0.35	0.003
Educational level (tertiary vs primary)	0.31	0.24	0.13 to 0.48	<0.001
Location (urban vs rural)	-0.02	-0.01	-0.13 to 0.10	0.782
Ethnicity (Chinese vs Malay)	-0.18	-0.12	-0.31 to -0.04	0.012
Ethnicity (Indian vs Malay)	-0.02	-0.02	-0.12 to 0.09	0.725
Ethnicity (others vs Malay)	-0.01	-0.01	-0.29 to 0.28	0.984

The dependent variable (global score) was square-root transformed to meet the assumptions of homoscedasticity. Model significance: F (12, 399)=17.318. P<0.001. Adjusted R^2^ = 0.323. B: unstandardised coefficient, β: standardised coefficient, CI: confidence interval, BMI: body mass index, GAD-7: Generalised Anxiety Disorder-7, PHQ-9: Patient Health Questionnaire-9.

Age was negatively associated with the EDE-Q scores (B=-0.01, 95% CI=-0.02 to -0.01, P<0.001). Higher BMI (B=0.02, 95% CI=0.02 to 0.03, P<0.001) and higher GAD-7 scores (B=0.05, 95% CI=0.03 to 0.08, P<0.001) were significant predictors of higher EDE-Q scores. The participants with secondary (B=0.21, 95% CI=0.07 to 0.35, P=0.003) and tertiary education (B=0.31, 95% CI=0.13 to 0.48, P<0.001) had significantly higher scores than those with no formal or primary education. In terms of ethnicity, the Chinese participants had lower EDE-Q scores than the Malay participants (B=-0.18, 95% CI=-0.31 to -0.04, P=0.012).

## Discussion

In this study, we found that the mean EDE-Q global score was relatively low among the participants with type 2 diabetes in Malaysia, in comparison with scores previously reported in patients with type 1 diabetes.^15^ Direct comparisons of the mean EDE-Q score in participants with type 2 diabetes are limited due to scarcity of research in this population. A systematic review showed that DEBs affected 1%-8% of adults with type 2 diabetes.^16^ The prevalence and characteristics of DEBs in patients with type 2 diabetes may be distinct from those among different populations, age groups and measurement tools. Our study reported a low level of DEBs among the participants compared with young adult Australian women.^17^ In our study, the study population was relatively older (mean age of 56 years), yet DEBs were observed in this group of participants. One contributing factor is that meal skipping and binge eating are commonly observed in patients with type 2 diabetes in Malaysia.^18^

Shape concern was the highest-scoring subscale in our study, suggesting that body image dissatisfaction is a consistent core feature of psychopathology.^19^ In our study, the adults with type 2 diabetes also showed a higher mean restraint subscale score. This could be due to the intentional limitations on food intake required for glycaemic control, driven by diabetes-specific dietary prescriptions and increased focus on weight after being diagnosed with type 2 diabetes.^20^

### Factors associated with DEBs

Our study revealed that the patients with a higher BMI had a higher EDE-Q score. This is consistent with existing evidence that individuals with overweight or obesity often exhibit higher levels of disordered-eating psychopathology.^21^ A higher BMI is linked with greater body dissatisfaction, which increases people’s concern about their shape and weight. Individuals with a high BMI also tend to engage in restrained eating or dieting behaviours, either voluntarily or as part of medical management.^22^

In our study, the participants with greater anxiety (GAD-7 score) had a higher EDE-Q score. A previous review has suggested that anxiety is significantly associated with different types of DEBs and EDs.^23^ Anxiety and DEBs can have a bidirectional relationship, where anxiety may drive disordered eating as a coping mechanism for diabetes-related distress, while the subsequent guilt and loss of control further intensify anxiety symptoms.^24^ Nevertheless, our study underscores the complex interaction between mental health, especially anxiety, and DEBs in patients with type 2 diabetes.

We found that younger age was negatively associated with the EDE-Q score. This is consistent with other reports showing that eating-disorder psychopathology tends to decline with increasing age.^25^ Younger individuals are exposed to sociocultural pressures around body shape and appearance (peer influence, social media and societal beauty ideals), which may lead to body dissatisfaction, shape/weight concerns and DEBs.^26^ Younger individuals are often in transitional life phases characterised by identity formation and insecurity, consequently increasing the prevalence of restrictive eating or binge eating. Young adults living with type 2 diabetes experience higher diabetes-related stress and lower diabetes self-efficacy, which complicate their mental health and diabetes management.^27^

In our study, a higher educational level was associated with a significantly higher EDE-Q score compared with only primary or no formal education. This finding aligns with previous review outcomes showing that a higher individual educational attainment is associated with increased risks for EDs.^28^ Psychosocial pressures of high-attainment environments may exacerbate DEBs in adults with type 2 diabetes. Individuals with EDs are often more educated than their peers. The pressure to maintain high standards, inherent in advanced educational tracks, may extend to body image, manifesting as heightened shape and weight concerns.^29^

For ethnicity, the Chinese participants reported lower EDE-Q scores than the Malay participants in our study population. This divergence likely reflects a combination of sociocultural and anthropometric differences in Malaysia. The Chinese population in Malaysia has been shown to have a lower mean BMI and a lower prevalence of obesity than the Malay population. Given that a higher BMI correlates with weight and shape concerns, the lower EDE-Q score observed in the Chinese participants may be partially explained by these distinct ethnic-specific BMI profiles.^30^

### Strengths and limitations

Our study covered participants from urban and rural primary care clinics. The adequate sample size and use of validated tools in measuring DEBs, depression and anxiety added research rigour to the study methodology. However, there are also several limitations in our study. First, the cross-sectional study design limits the ability to establish causal relationships between the independent variables and DEBs. Second, the use of convenience sampling in this study may have introduced selection bias. Third, the identification of DEBs, depression and anxiety was based solely on scores from screening questionnaires (EDE-Q, PHQ-9 and GAD-7) and was not confirmed through a definitive diagnostic assessment by a psychiatrist. Fourth, our study excluded participants lacking proficiency in either Bahasa Malaysia or English, potentially limiting the generalisability of the results to the full range of linguistic diversity within the Malaysian population with type 2 diabetes.

### Clinical and research implications

Given the association of the EDE-Q score with younger age, higher BMI and greater anxiety, opportunistic screening could identify patients at a high risk of DEBs. The higher mean scores for the shape concern and restraint subscales show a need for better dietary education to balance the medical need for glycaemic control with the potential of triggering pathological restrictive eating. Managing patients with concurrent DEBs and type 2 diabetes effectively may require an integrated care model. Standard medical reviews can be insufficient to address the intersection of metabolic and eating psychopathologies. A multidisciplinary team consisting of primary care physicians, psychologists and dietitians can collaboratively identify and manage these cases. Future research should focus on longitudinal designs to better understand how anxiety and DEBs contribute to long-term diabetes outcomes such as HbA1c levels. Qualitative approaches would be useful to explore the cultural context and lived experiences of DEBs among individuals with type 2 diabetes in Malaysia’s diverse population.

## Conclusion

While the overall level of DEBs in adults with type 2 diabetes is low, suggesting that formal routine screening may not be warranted, clinical vigilance remains crucial for specific high-risk vulnerable groups. Clinicians should consider opportunistic monitoring of eating behaviours during routine care for patients with younger age, higher BMI, greater anxiety and higher educational level and psychological health interventions for more holistic diabetes care.

## Data sharing statement

Data are available upon request from the corresponding author.

**How does this paper make a difference in general practice?**

- Disordered eating behaviours (DEBs) significantly impair metabolic control, yet research on these behaviours in adults with type 2 diabetes remains limited.
- While the overall DEB level is relatively low, shape concern and restrictive eating are prominent.
- DEBs are independently associated with younger age, higher BMI, greater anxiety and higher educational level.
- Clinicians should adopt opportunistic screening strategies for high-risk subgroups to provide targeted interventions that improve both psychological and glycaemic outcomes, such as balanced dietary education.
